# Breeding migrations by bighorn sheep males are driven by mating opportunities

**DOI:** 10.1002/ece3.8692

**Published:** 2022-03-06

**Authors:** Roxane Lassis, Marco Festa‐Bianchet, Fanie Pelletier

**Affiliations:** ^1^ 7321 Département de biologie et Centre d’Études Nordiques Université de Sherbrooke Sherbrooke Quebec Canada

**Keywords:** Bighorn sheep, breeding migration, breeding sex ratio, metapopulation, trophy hunting

## Abstract

In some species where male mating success largely depends on intrasexual competition, males can adopt migratory or resident strategies to seek breeding opportunities. The resulting mixture of resident and migrant tactics within a population can have important ecological, genetic, and evolutionary consequences for metapopulations. Bighorn sheep *Ovis canadensis* males establish a linear dominance hierarchy that influences their mating tactics. Some males perform breeding migrations during the pre‐rut and rut to seek mating opportunities, but little is known about these seasonal movements. We analyzed presence/absence data for 62 marked bighorn males during six mating seasons (20–32 males/year) in the Sheep River Provincial Park, Alberta, Canada, where hunting was not allowed. On average, about half of males left their natal population to rut elsewhere. The proportion of males leaving (yearly range 15%–69%) increased as the number of resident mature males increased and the populational sex ratio decreased, with fewer females during the pre‐rut. Among those leaving the park, 24% did so in October, while the trophy sheep hunting season was open. Detailed monitoring of breeding migrations in protected populations could inform management strategies to limit evolutionary impacts of hunting, which can alter size‐dependent mortality and create artificial pressures driving changes on heritable traits.

## Graphical abstract

In bighorn sheep, *Ovis canadensis*, some males undertake breeding migrations during the pre‐rut and rut periods to seek mating opportunities. We explored the individual and populational determinants of male migratory rutting tactics and the timing of these movements in the fall. As male undertaking breeding migrations are more at risk of getting harvested, especially when they move out of protected areas, our results have implications for wild sheep management as gene flow may be reduced and insufficient to limit hunting‐induced artificial selection on horn size.

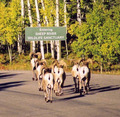

## 1 INTRODUCTION

Understanding animal movements is crucial to the study of gene flow, evolution, and the conservation of wildlife because of their profound ecological and evolutionary consequences (Dieckmann et al., 1999; Garant et al., 2007). Movement of individuals can shape local and global diversity, influence population dynamics, community structure, and evolutionary processes. By affecting gene flow and connectivity between populations, movements may drive local adaptation, speciation, and the evolution of life‐history traits (Dieckmann et al., 1999; Garant et al., 2007; Hanski, 1999; Nathan et al., 2008).

The term “migration” encompasses a variety of movement behaviors, from daily vertical shifts of fish to interpolar seasonal flights of birds (Fontane cisco, *Coregonus fontanae*, Mehner 2014, and Arctic tern, *Sterna paradisea*, Mander 2021, reviewed by Chapman et al., 2011), that vary among species, populations, and individuals (Dingle, 1996). Partial migration occurs when only a fraction of the population migrates, distinguishing migrant from nonmigrant individuals (Chapman et al., 2011a; Dingle, 1996). Partial migration has been documented in invertebrates, fish, birds, and mammals (Chapman et al., 2011a, 2011). It can be promoted by differences among individuals in several factors including predation risk, learning, or competition for resources including breeding opportunities (Chapman et al., 2011; Jesmer et al., 2018; Merkle et al., 2019).

In polygynous species where male mating success largely depends on intrasexual competition, breeding migration can be a part of a mating tactic, leading seasonally to variability in movements of males seeking mates (Andersson & Iwasa, 1996; Chapman et al., 2011a; Shaw & Levin, 2011). Some males may temporarily leave their natal population to seek mates elsewhere while others remain to attempt to breed within their natal population (Chapman et al., 2011; Shaw & Levin, 2011). Male breeding migrations have been documented in multiple species (Shaw & Levin, 2011), but the underlying ecological and evolutionary processes are poorly understood. Breeding migration may impose energetic costs from travelling and increase mortality risks if they involve traversing unsafe habitats. Therefore, they may be governed by spatial differences in mating opportunities and fitness trade‐offs (Chapman et al., 2011a, 2011; Shaw & Levin, 2011).

Bighorn sheep, *Ovis canadensis*, show multiple forms of migration including local shifts within home ranges, migrations between seasonal home ranges, and rare natal dispersal (Geist, 1971; Hogg, 2000). For instance, in a population in Sheep River, Alberta, sheep seasonally migrate between a winter range at elevations of 1420–1740 m and an alpine summer range approximately 12–15 km to the west at elevations of 1800–2550 m (Festa‐Bianchet, 1986a, 1988). Mountain sheep are gregarious but form sexually segregated groups (Ruckstuhl, 1998). Seasonal migrations in females are mostly driven by spatial differences in forage availability and quality, although the timing may be affected by predator avoidance around parturition (Festa‐Bianchet, 1986a, 1988; Geist, 1971). Although males can also migrate in spring‐summer to seek better seasonal forage, in the 4–6 weeks preceding the rut some males perform breeding migrations (Hogg & Forbes, 1997; Pelletier et al., 2006). During the pre‐rut, males often gather in congregations that appear to have a social significance, as agonistic interactions reinforce a linear dominance hierarchy (Festa‐Bianchet, 1986a; Geist, 1971; Pelletier & Festa‐Bianchet, 2006). Hogg (2000) hypothesized that males may use information gathered in the pre‐rut congregation to assess whether to leave or stay for the rut based on their dominance rank and the number of available females in their natal population. In the Sheep River population, Festa‐Bianchet, 1986a, 1986b, 1991) found that 20%–50% of males left to rut elsewhere, and Hogg (2000) reported that most males which left joined other ewe groups 20–25 km away. However, little is known about the precise timing and the factors influencing these male movements (Pelletier et al., 2014), which could be instrumental in limiting inbreeding and maintaining genetic diversity within the metapopulation. These considerations are particularly important given that in bighorn sheep natal dispersal is extremely rare (Festa‐Bianchet, 1986a).

In addition to fitness trade‐offs, breeding migrations could expose bighorn males to anthropogenic pressures, including trophy hunting that selectively targets large‐horned males outside protected areas (Festa‐Bianchet, 2017). Several long‐term studies have reported morphological changes consistent with hunting‐induced evolution of smaller horn size (Douhard et al., 2016; Festa‐Bianchet et al., 2014; Hengeveld & Festa‐Bianchet, 2016; Pigeon et al., 2016). Evolutionary changes in horn growth are expected under a restricted set of conditions (Festa‐Bianchet, 2017) and require intense selective harvest of young but large‐horned males (LaSharr et al., 2019; Morrissey et al., 2021). The ecological and evolutionary consequences of selective hunting may be mitigated through genetic rescue if unselected males from protected areas move to breed in hunted areas (Dunlop et al., 2009; Tenhumberg et al., 2004). Two studies of hunted bighorn sheep in Alberta, Canada, found limited evidence of genetic rescue from harvest refuges(Pelletier et al., 2014; Poisson et al., 2020). These studies suggested that some males originating from populations in protected areas are harvested in late October. The frequency and timing of male breeding migrations from protected areas may affect their probability to be killed and therefore to provide a genetic rescue in exploited populations (Pelletier et al., 2014).

The goal of this study was to investigate how individual and demographic characteristics affect the probability of breeding migrations by bighorn males. We also wanted to document the timing and frequency of migrations, as they influence hunting vulnerability of males leaving protected areas. We hypothesized that individual and populational characteristics will influence the behavior of adult males, with breeding migrations adopted by those with lower breeding opportunities in their natal population. We therefore expected that young to middle‐aged and subordinates mature males will migrate, while older and dominant males will breed within their population of origin (see also Hogg, 2000). We also expected that demography should affect male movement. Based on Hogg (2000), we predicted that more males would undertake breeding migrations as the number of competitors increased, when male age structure was older and when the pre‐rut population sex ratio suggested low availability of females. To test these predictions, we analyzed the presence/absence data for 62 marked bighorn males during six mating seasons (20–32 males/year) in Sheep River Provincial Park, where local conditions allow an unusually high efficiency of detection of marked sheep.

## 2 MATERIALS AND METHODS

### 2.1 Study area and population

We analyzed data collected in 2000–2005 from marked known‐age bighorn sheep in the Sheep River Provincial Park (50°40′N, 114°35W, elevation 1450–1700 m), Alberta, Canada. The Park (60 km^2^) included about 12 km^2^ of sheep habitat with a shale canyon incised by the Sheep River, grassy slopes, steep hillsides, and cliffs (Festa‐Bianchet, 1986a). The data collection protocol was approved by the Animal Care Committee of the Université de Sherbrooke, an affiliate of the Canadian Council on Animal Care. Since 1981, more than 90% of the sheep were captured using drugs delivered by a dart gun or a corral trap baited with salt and marked with unique combinations of ear tags (Festa‐Bianchet, 1986b; Festa‐Bianchet & Jorgenson, 1985; Hogg & Forbes, 1997). Most males resident in the Sheep River population were marked as lambs, but several nonresident males that temporally immigrated for the mating season were unmarked. The latter (representing 0–26% of males each year, Figure S1, Table S1) were removed from analyses because we had incomplete information on these individuals that were recognized within each season from horn shape and size and coat characteristics. Nearly all resident males were known to be born from resident mothers and were first captured at 4–7 months of age, except for 5 permanent immigrants that were marked aged 1 or 2 years (accounting for 0–8% of resident males each year). We excluded 10 resident males in the year of their death, because they died before the start of the rut.

In Autumn, males form a pre‐rut congregation and reinforce a linear dominance hierarchy before the rut, which occurs in late November and early December (Hogg & Forbes, 1997; Pelletier et al., 2006). Between 2000 and 2005, most days from mid‐September to mid‐December, two to five observers searched the Park on foot to locate groups of sheep and monitor their behavior (Pelletier & Festa‐Bianchet, 2004, 2006; Pelletier et al., 2006). During daily searches, identities of all individuals seen were recorded. Bighorn sheep in this population are easy to find because most of their habitat is visible from a road, it is open with high visibility and sheep are gregarious, so that most of the population is typically in a few large groups. For example, efficiency in finding marked females during winter was 97%: on average, only 3% were missed during single‐day searches of the winter range (Festa‐Bianchet, 1986b). From these searches of the entire winter range, we extracted daily presence–absence of marked males from the 1st of October to the end of the observation season (range December 3–18, Table S2). The annual study period lasted on average 72 days (range 64–79 days), and on average searches were conducted in 90% of days (range 84–99%). We considered the rut to begin the day the first estrus was observed (Pelletier et al., 2006), on average November 22 ± 3 days. During this study, the population averaged 27 (yearly range 19–39) females and 26 (range 20–32) resident males. Each year, 67–97% of females aged 2 years or older were seen in estrus, suggesting that most of the mating season was documented (Pelletier et al., 2006). Survival over the rut was known for males that were seen in mid‐December or during subsequent fieldwork, typically beginning the following May. Some males were known to have died because they were found dead or reported shot by hunters, who must register harvested trophy sheep. Capture–mark–recapture analyses of this population estimated the resighting rate for males at over 95% (Loison et al., 1999).

### 2.2 Demographic parameters

We calculated demographic parameters for the population during the pre‐rut, from the 1st of October to the first estrus observed. We estimated three variables known to affect male reproductive success (Martin et al., 2016): the number of competing males aged 2 years or older; male age structure, defined as the ratio of males aged 2–4 years over the number of males 2 years and older. We chose 5 years as a threshold because males younger than 5 years generally cannot adopt the highly successful mating tactic of tending (Pelletier & Festa‐Bianchet, 2006). Population sex ratio was defined as the number of females 2 years or older over the number of males 2 years or older in the pre‐rut.

### 2.3 Individual characteristics

In addition to age, we used standardized social rank for each male from Pelletier and Festa‐Bianchet (2004). Each year, matrices were constructed based on six types of social interactions: front kick, horn rubbing, mount, frontal clash, butt and noncontact displacements (Hass & Jenni, 1991; Hogg, 1987; Pelletier & Festa‐Bianchet, 2004). The method proposed by de Vries (1998) was used to order males based on the outcome of dyadic encounters. The linearity of the hierarchy was estimated with the Landau index implemented in Matman 1.0 (Landau, 1951; Pelletier & Festa‐Bianchet, 2004; de Vries, 1995). On average, 549 interactions were recorded per year (range 261–741) involving 56% of possible male dyads (range 46–67%). Only males aged 2 years and older seen interacting with at least 5 other males were included in the interaction matrix. Social rank was standardized annually by the total number of rams and ranged from 1 to 0, where 1 is the top dominance position. Yearling males were assigned a rank of 0 because they rarely interacted with other males. For more details on the methods, see Pelletier and Festa‐Bianchet (2004).

### 2.4 Statistical analyses

We investigated how age, rank, and demography affected the probability of breeding migration by fitting Bayesian generalized linear mixed models. The proportion of males leaving to rut elsewhere and timing of migrations were assessed by calculating the annual proportion of migrants and the date of last annual observation of each migrant. Resident males present during the pre‐rut and absent during the rut were considered “migrants,” while “sedentary” males were those present during both periods.

**FIGURE 1:**
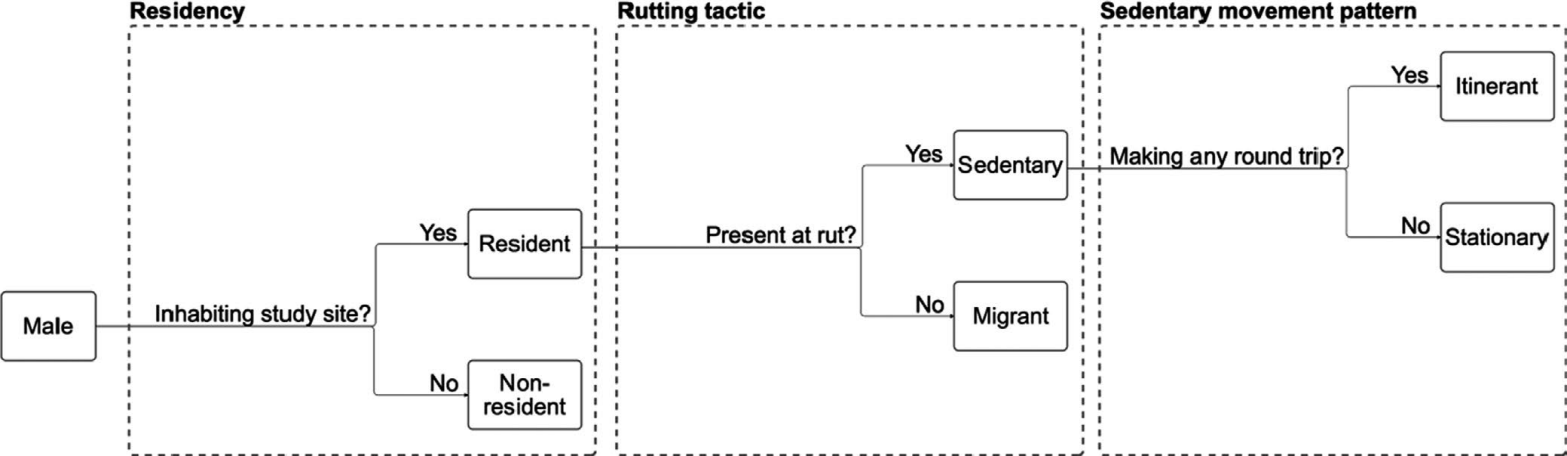
Classification of rutting tactics based on migratory behavior of male bighorn sheep

We further explored movement patterns of sedentary males using the distribution of consecutive days they were missed during searches. Of these apparent absences, 75% lasted 1–3 consecutive days and 90% 1–6 consecutive days (Figure S2). We used this 90‐percentile as a threshold and classified as temporary movements absences longer than 6 days. We refer to these temporary movements as “round trips” because males were seen again after a few days to a few weeks and were present for the rut. About one‐quarter of temporary round trips were confirmed by direct observations of a male leaving the park and heading to the mountains to the west. Sedentary males making at least one round trip during the pre‐rut or the rut were referred to as “itinerants” and the rest as “stationary” (Figure 1). Seven sedentary males were excluded from analysis of round trips in the year they died, because they were last seen during the rut and then reported dead.

As male age and social rank were correlated (Pearson correlation *r* = .86), their effects were modeled separately. We fitted B‐splines with degree up to two with the “bs” function in the SPLINES package (Perperoglou et al., 2019) to determine the appropriate polynomial degree for individual parameters. Models included the linear and quadratic terms of each individual parameter, the three demographic parameters and their interactions. We included the number of searches each year as an explanatory variable to account for variation in sampling effort. We also included male identity and year as random variables to control for pseudo‐replication and annual environmental effects such as events of intense cougar (*Puma concolor*) predation (Bourbeau‐Lemieux et al., 2011).

All statistical analyses were implemented in R version 4.0.5 (R Core Team, 2021). Models were fitted using Bernoulli Bayesian regressions with a logit link using the “BRMS” library (Bürkner, 2017). All models were run with 30 000 iterations for a warm‐up of 15,000 iterations, a Rhat of 1. The lowest effective sample size (ESS) was acceptable, and the chains were inspected visually. Among all candidate models, including different combinations of individual and populational parameters (Table S3), we selected the model with the lowest leave‐one‐out cross‐validation information criterion (LOOIC, Bürkner, 2017; Vehtari et al., 2015). We present estimates from the selected model.

## 3 RESULTS

### 3.1 Rutting tactics: migratory or sedentary

**FIGURE 2:**
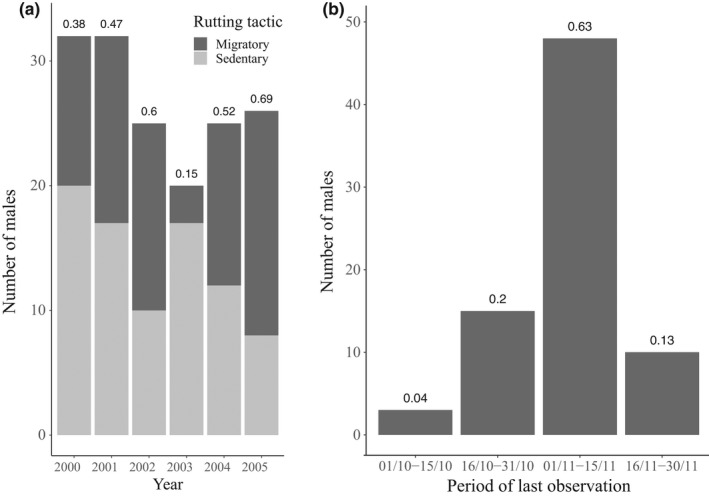
(a) Annual rutting tactics of resident bighorn males one year and older. Migrant males left the study site to rut elsewhere. Sedentary males were present for the rut. Numbers on top of bars represent annual proportions of breeding migrants. (b) Number of migrant departures per period with associated proportions on top of bars, based on dates of last observation by half‐month, between the 1st of October and the end of the seasonal study period, Sheep River Provincial Park, 2000–2005, Alberta, Canada

**FIGURE 3:**
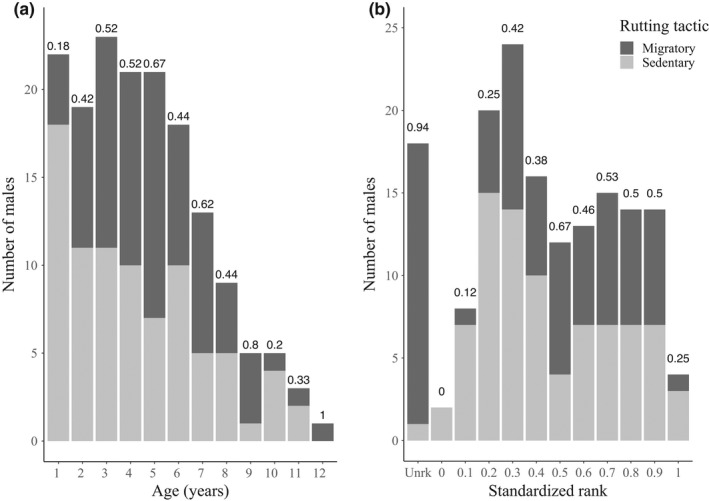
Distribution of (a) age and (b) standardized rank (rank 1 is most dominant) of resident bighorn males by rutting tactic (dark grey for “Migrant,” light gray for “Sedentary”), Sheep River Provincial Park, 2000–2005, Alberta, Canada. Labels on top of bars represent proportions of migrants in each age or rank class. Rank class “Unrk” includes males of unknown dominance rank

The proportion of males adopting a migratory tactic, leaving the study site to rut elsewhere, averaged 47% (yearly range 15–69%, Figure 2a). While 63% of departures for breeding migrations occurred in the first 15 days of November, 24% were in October (Figure 2b). Most migrants were of mid‐age and mid‐rank (Figure 3).

**FIGURE 4:**
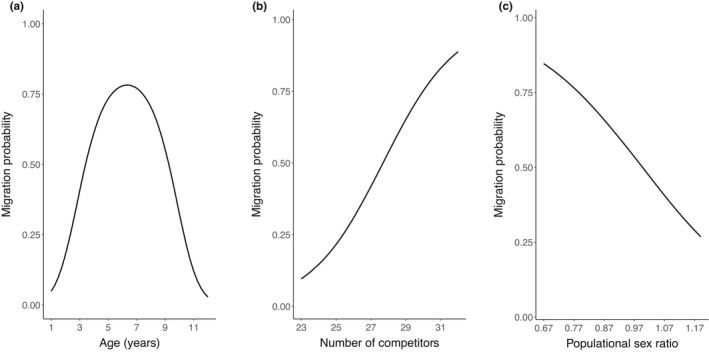
Effects of (a) age, (b) number of competitors (males aged 2 years or older in the pre‐rut), and (c) populational sex ratio (females 2 years or older over males 2 years or older in the pre‐rut) on the probability that resident bighorn males adopted a migratory rutting tactic, Sheep River Provincial Park, 2000–2005, Alberta, Canada. Regressions lines represent estimates from the final model with other nonfocal variables set to their mean value (e.g., age at 5 years in panels b and c). Points represent observed proportions of migrants

The selected final model for factors influencing breeding migrations included fixed quadratic effects of age and linear effects of number of competitors and populational sex ratio (Table S4). The final model estimated a concave relationship between male age and probability to migrate, peaking at 78% [95% CI: 0.45, 0.95] at age 6.25 years (Figure 4a, Table 1). The proportion of migrants increased with number of competitors (Figure 4b, Table 1) and decreased with populational sex ratio (Figure 4c, Table 1). The final model estimated a near‐50% probability to migrate for males aged 5 years in a population with 27.5 competitors (52% [95% CI: 0.16, 0.82], Figure 4b), or with a population sex ratio of 1.11 females per male (51% [95% CI: 0.13, 0.87], Figure 4c).

**TABLE 1:** Coefficients (on the logit scale) with corresponding 95% confidence intervals, bulk effective sample size (ESS), and tail ESS of fixed variables of the final model assessing the determinants of the probability of migratory rutting tactic, Sheep River Provincial Park, 2000–2005, Alberta, Canada. Estimates are from the final model including male identity and year as random variables

Variable	Coefficient	95% CI	Bulk ESS	Tail ESS
Intercept	−10.79	[−30.74; 8.86]	17638	13599
Age	9.02	[4.00; 15.35]	15616	16612
Age^2^	−0.61	[−4.58; 3.19]	18246	19050
Number of competitors	0.49	[0.10; 0.92]	15363	14825
Populational sex ratio	−5.31	[−11.37; 0.39]	16154	12051
Sampling effort	−0.03	[−0.23; 0.16]	17712	12181

When fitted with a reduced data set including only males with information on both age and social rank, models including rank performed less well than those with age in explaining the probability of breeding migration (Table S5). Among those with social rank as individual parameter, the model with the lowest LOOIC had fixed quadratic effects of rank and fixed linear effects of number of competitors and of populational sex ratio (Figure S3, Table S6). This final model estimated a maximal probability to migrate (69% [95% CI: 0.33, 0.92], Figure S3a) for males with a rank of 0.65. Therefore, top‐ranking and very subordinate males were less likely to undertake breeding migrations than middle‐ranking males.

### 3.2 Movement patterns of itinerant residents

**FIGURE 5:**
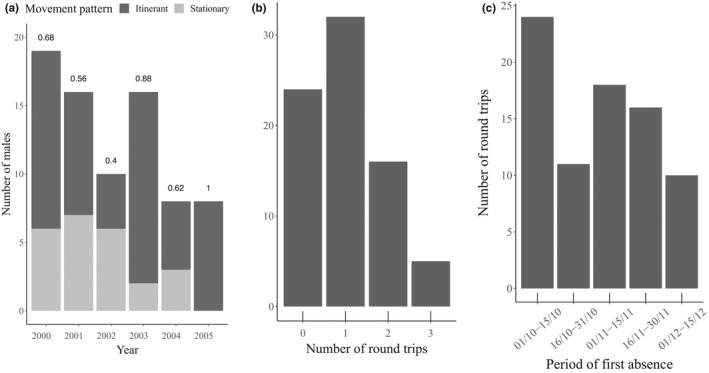
(a) Annual distribution of movement patterns of resident bighorn males. “Itinerant” males left at least once, “Stationary” males stayed in the natal population. Labels on top of bars represent annual proportions of itinerants), (b) number of round trips, defined as absences longer than 6 days, by individual‐year, and (c) half‐month of first day of round trips by individual‐year, for resident individuals that returned for the rut, between the 1st of October and the end of the seasonal study period, Sheep River Provincial Park, 2000–2005, Alberta, Canada

The annual proportion of itinerants among sedentary males averaged 69% (range 40–100%, Figure 5a). Of these, 42% undertook a single round‐trip, and 27% made 2 or 3 trips (Figure 5b). Departures of round trips were detected from early October to mid‐December, with 30% during the first 15 days of October (Figure 5c).

## 4 DISCUSSION

Our study supported the hypothesis proposed by Hogg (2000) that males use individual and populational cues gathered during the pre‐rut congregation to assess whether to adopt a migratory or a sedentary breeding tactic. As expected, middle‐aged and mid‐ranked males had the highest probabilities to leave the population winter range during the pre‐rut and the rut, suggesting a potential breeding migration. Though rams reach sexual maturity at around 1.5 years, they are usually prevented from breeding by dominant older males until they are 3 years or older (Ritchot et al., 2021). Therefore, young adult males likely had low propensity to migrate because they had very low breeding opportunities in any ewe group. Male reproductive success increases nonlinearly with age and distribution of paternities is skewed in favor of a few top‐ranking males (Coltman et al., 2002; Hogg & Forbes, 1997; Pelletier & Festa‐Bianchet, 2006). Consequently, older and dominant males often stay within their natal population where they have a high expected mating success. Contrary to our expectations, age better explained variation in migratory behavior than social rank, which is a strong predictor of yearly male mating success (Pelletier & Festa‐Bianchet, 2006). Nevertheless, age and rank being correlated, these results may arise from age‐dependent mating tactics (Hogg & Forbes, 1997; Pelletier & Festa‐Bianchet, 2006; Pelletier et al., 2006). Subordinates and middle‐aged rams are forced by dominants to use alternative mating tactics (coursing instead of tending) whose effectiveness depends on the number of competitors and the availability of females (Martin et al., 2016; Pelletier & Festa‐Bianchet, 2006; Pelletier et al., 2006). The probability of breeding migration increased with the number of adult males and decreased with populational sex ratio in the pre‐rut, suggesting that males were more likely to migrate when their local mating opportunities were low.

In addition to migrants, some sedentary males appeared to undertake temporary movements but returned for the rut. We analyzed data on presence/absence in the winter range, but we did not know the whereabouts of breeding migrants or males undertaking round trips when we did not see them. Earlier work with radio‐collared males in this population, however, found that in October–December males that left the winter range were relocated in areas with other wintering ewe groups, or in transit between these areas (Festa‐Bianchet, 1986a; Hogg, 2000). Males which move during the mating season may explore breeding opportunities in nearby groups of females. Temporary absences could also reflect a decreased participation in rutting activities by sedentary males that exhausted metabolic reserves (Pelletier, 2005; Pelletier & Festa‐Bianchet, 2004). By temporarily resting or foraging elsewhere, males could compensate the energetic costs of agonistic encounters during the pre‐rut and compete for mates during the rut.

Several studies have shown that migratory patterns of large herbivores are affected by forage quality and quantity, weather, and predation (Merkle et al., 2016; Middleton et al., 2013). Male breeding migrations could also have been driven by environmental constraints, such as deep snow, but were unlikely to be affected by forage quality as by October forage is dormant in all seasonal ranges. We partially controlled for possible environmental variation by including year as a random variable.

Although we did not have GPS trackers on these animals, our findings rely on an exceptionally detailed data set of daily behavior of males during the pre‐rut and the breeding season. For example, in 2000–2002, we collected an average of 28.2 h of observations for each male during the pre‐rut and 27.3 h during the rut (Pelletier, 2005). When resident females were in the winter range, we found an average of 97% of marked ones in each daily search (Festa‐Bianchet, 1986b). It is extremely unlikely that we would miss a male for 6 days if it was in the winter range. On average, 69% of the males classified as migrants were present the following mating season (Table S7); however, there is an uncertainty as to the fate of individuals that were not accounted for and could have died or permanently emigrated. Due to incomplete information, our approach could not provide a highly accurate index of migratory propensity. A more precise tracking of individuals is necessary to improve our current knowledge on male movement and space use.

The timing of seasonal movements can be decisive for male survival and reproduction in harvested species (Dunlop et al., 2009; Puckett & Eggleston, 2016). Males that leave a protected area during the hunting season are exposed to mortality risk from hunting. In the Sheep River population, 24% of male breeding migrations and 35% of round trips started in October. The trophy sheep hunt in Alberta lasts until the end of October. In exploited areas of the province, quota‐free trophy hunting targets males whose horn curl describes 4/5 of a curl or more, a status usually attained at 4–7 years of age depending on horn length and shape (Festa‐Bianchet et al., 2014). Males leaving protected areas in October are vulnerable to hunting. For example, three males in 2001 were poached and two in 2005 were legally shot outside of the park, representing respectively about 8% and 5% of the yearly number of males. Overall, from 1982 to 2005, 23 males were shot in October after they left the protected area. Other studies in Alberta have shown that during the pre‐rut and rut, some mature males exit protected areas such as National Parks and are harvested late in the hunting season (Pelletier et al., 2014; Poisson et al., 2020). The removal of migrant males before the mating season may reduce gene flow at the metapopulation level and limit the potential for genetic rescue to buffer hunting‐induced artificial selection. For instance, in our study population between 2000 and 2005, the annual permanent immigration rate of males averaged 3% (yearly range: 0–6%). To limit inbreeding and maintain genetic and phenotypic diversity (Pelletier et al., 2014; Poisson et al., 2020), hunting regulations should seek to reduce mortality of migrant breeders and favor genetic rescue by closing the hunting season in mid‐October. Monitoring the movement of animals from protected areas through GPS telemetry would provide useful information for long‐term conservation strategies of harvested species. Planning decisions are increasingly informed by studies of animal movements and landscape connectivity (e.g. caribou, *Rangifer tarandus*, Fullman et al., 2017). Sustainable management and conservation of wildlife require considerations of both spatial and temporal components of animal movements between protected and exploited areas to maintain gene flow and reduce harvest of animals from protected areas.

## CONFLICT OF INTEREST

The authors declare there are no competing interests.

## AUTHOR CONTRIBUTIONS

**Roxane Lassis:** Formal analysis (lead); Methodology (equal); Writing – original draft (lead); Writing – review & editing (equal). **Marco Festa‐Bianchet:** Methodology (equal); Writing – review & editing (equal). **Fanie Pelletier:** Conceptualization (lead); Methodology (equal); Writing – review & editing (equal).

## Supporting information

Supplementary Material

## Data Availability

Data available from the Dryad Digital Repository: https://doi.org/10.5061/dryad.x0k6djhmh.

## References

[ece38692-bib-0001] Andersson, M. , & Iwasa, Y. (1996). Sexual selection. Trends in Ecology & Evolution, 11(2), 53–58. 10.1016/0169-5347(96)81042-1 21237761

[ece38692-bib-0004] Bourbeau‐Lemieux, A. , Festa‐Bianchet, M. , Gaillard, J.‐M. , & Pelletier, F. (2011). Predator‐driven component Allee effects in a wild ungulate. Ecology Letters, 14(4), 358–363. 10.1111/j.1461-0248.2011.01595.x 21320261

[ece38692-bib-0005] Bürkner, P.‐C. (2017). Advanced Bayesian Multilevel Modeling with the R Package brms. The R Journal, 10(1), 395–411. Technische Universitaet Wien. Retrieved from https://arxiv.org/abs/1705.11123v2 [accessed 26 July 2021]

[ece38692-bib-0006] Chapman, B. , Brönmark, C. , Nilsson, J.‐Å. , & Hansson, L.‐A. (2011a). Partial migration: An introduction. Oikos, 120(12), 1761–1763. 10.1111/j.1600-0706.2011.20070.x

[ece38692-bib-0007] Chapman, B. B. , Brönmark, C. , Nilsson, J.‐Å. , & Hansson, L. (2011b). The ecology and evolution of partial migration. Oikos, 120(12), 1764–1775.

[ece38692-bib-0008] Coltman, D. W. , Festa‐Bianchet, M. , Jorgenson, J. T. , & Strobeck, C. (2002). Age‐dependent sexual selection in bighorn rams. Proceedings of the Royal Society B‐Biological Sciences, 269(1487), 165–172. 10.1098/rspb.2001.1851 PMC1690873 11798432

[ece38692-bib-0009] de Vries, H. (1995). An improved test of linearity in dominance hierarchies containing unknown or tied relationships. Animal Behavior, 50(5), 1375–1389. 10.1016/0003-3472(95)80053-0

[ece38692-bib-0010] de Vries, H. (1998). Finding a dominance order most consistent with a linear hierarchy: a new procedure and review. Animal Behavior, 55(4), 827–843. 10.1006/anbe.1997.0708 9632471

[ece38692-bib-0011] Dieckmann, U. , O'Hara, B. , & Weisser, W. (1999). The evolutionary ecology of dispersal. Trends in Ecology & Evolution, 14(3), 88–90. 10.1016/S0169-5347(98)01571-7

[ece38692-bib-0012] Dingle, H. (1996). Migration: The Biology of Life on the Move ‐ Oxford Scholarship. Retrieved from https://oxford.universitypressscholarship.com/view/ 10.1093/acprof:oso/9780199640386.001.0001/acprof-9780199640386 [accessed 6 July 2021]

[ece38692-bib-0013] Douhard, M. , Festa‐Bianchet, M. , Pelletier, F. , Gaillard, J. M. , & Bonenfant, C. (2016). Changes in horn size of Stone’s sheep over four decades correlate with trophy hunting pressure. Ecological Applications, 26(1), 309–321. 10.1890/14-1461.1/suppinfo 27039527

[ece38692-bib-0014] Dunlop, E. S. , Baskett, M. L. , Heino, M. , & Dieckmann, U. (2009). Propensity of marine reserves to reduce the evolutionary effects of fishing in a migratory species. Evolutionary Applications, 2(3), 371–393. 10.1111/j.1752-4571.2009.00089.x 25567887 PMC3352486

[ece38692-bib-0015] Festa‐Bianchet, M. (1986a). Site fidelity and seasonal range use by bighorn rams. Canadian Journal of Zoology, 64(10), 2126–2132. 10.1139/z86-326

[ece38692-bib-0016] Festa‐Bianchet, M. (1986b). Seasonal dispersion of overlapping mountain sheep ewe groups. The Journal of Wildlife Management, 50(2), 325–330. 10.2307/3801922

[ece38692-bib-0017] Festa‐Bianchet, M. (1988). Seasonal range selection in bighorn sheep: conflicts between forage quality, forage quantity, and predator avoidance. Oecologia, 75(4), 580–586. 10.1007/BF00776423 28312434

[ece38692-bib-0058] Festa‐Bianchet, M. (1991). The social system of bighorn sheep: grouping patterns, kinship and female dominance rank. Animal Behaviour, 42(1), 71–82. 10.1016/S0003-3472(05)80607-4

[ece38692-bib-0018] Festa‐Bianchet, M. (2017). When does selective hunting select, how can we tell, and what should we do about it? Mammal Review, 47(1), 76–81. 10.1111/mam.12078

[ece38692-bib-0019] Festa‐Bianchet, M. , & Jorgenson, J. T. (1985). Use of xylazine and ketamine to immobilize bighorn sheep in Alberta. Journal of Wildlife Management, 49(1), 162. 10.2307/3801864

[ece38692-bib-0020] Festa‐Bianchet, M. , Pelletier, F. , Jorgenson, J. T. , Feder, C. , & Hubbs, A. (2014). Decrease in horn size and increase in age of trophy sheep in Alberta over 37 years. Journal of Wildlife Management, 78(1), 133–141. 10.1002/jwmg.644

[ece38692-bib-0021] Fullman, T. J. , Joly, K. , & Ackerman, A. (2017). Effects of environmental features and sport hunting on caribou migration in northwestern Alaska. Movement Ecology, 5(1), 1–11. 10.1186/s40462-017-0095-z 28270913 PMC5331706

[ece38692-bib-0022] Garant, D. , Forde, S. E. , & Hendry, A. P. (2007). The multifarious effects of dispersal and gene flow on contemporary adaptation. Functional Ecology, 21(3), 434–443. 10.1111/j.1365-2435.2006.01228.x

[ece38692-bib-0023] Geist, V. (1971). Mountain sheep. A study in behavior and evolution. Mt. sheep. University of Chicago Press.

[ece38692-bib-0024] Hanski, I. (1999 ). Metapopulation Ecology ‐ Ilkka Hanski, Professor in the Department of Ecology and Systematics Ilkka Hanski ‐ Google Books. Retrieved from https://books.google.ca/books?hl=en&lr=&id=jsk4Nt_8X8sC&oi=fnd&pg=PA1&dq=Hanski+I+(1999)+Metapopulation+Ecology&ots=gDYi0nJX2G&sig=E9hQeQG0Y4UlGfgvtwKhYbzQ3cQ#v=onepage&q=HanskiI(1999)MetapopulationEcology&f=false (accessed 11 July 2021).

[ece38692-bib-0026] Hass, C. C. , & Jenni, D. A. (1991). Structure and ontogeny of dominance relationships among bighorn rams. Canadian Journal of Zoology, 69(2), 471–476. 10.1139/z91-073

[ece38692-bib-0027] Hengeveld, P. E. , & Festa‐bianchet, M. (2016). Harvest regulations and artificial selection on horn size in male bighorn sheep. Journal of Wildlife Management, 75(1), 189–197. 10.1002/jwmg.l4

[ece38692-bib-0028] Hogg, J. T. (1987). Intrasexual competition and mate choice in rocky mountain bighorn sheep. Ethology, 75(2), 119–144. 10.1111/j.1439-0310.1987.tb00647.x

[ece38692-bib-0029] Hogg, J. T. (2000). Mating systems and conservation at large spatial scales. In M. Apollonio , M. Festa‐Bianchet , & D. Mainardi (Eds.), Vertebrate mating systems (pp. 214–252). World Scientific. 10.1142/9789812793584_0010

[ece38692-bib-0030] Hogg, J. T. , & Forbes, S. H. (1997). Mating in bighorn sheep: frequent male reproduction via a high‐risk "unconventional" tactic. Behavioral Ecology and Sociobiology, 41(1), 33–48. 10.1007/s002650050361

[ece38692-bib-0031] Jesmer, B. R. , Merkle, J. A. , Goheen, J. R. , Aikens, E. O. , Beck, J. L. , Courtemanch, A. B. , Hurley, M. A. , McWhirter, D. E. , Miyasaki, H. M. , Monteith, K. L. , & Kauffman, M. J. (2018). Is ungulate migration culturally transmitted? Evidence of social learning from translocated animals. Science, 361(6406), 1023–1025. 10.1126/science.aat0985 30190405

[ece38692-bib-0033] Landau, H. G. (1951). On dominance relations and the structure of animal societies: I. Effect of inherent characteristics. The Bulletin of Mathematical Biophysics, 13(1), 1–19. 10.1007/BF02478336

[ece38692-bib-0034] LaSharr, T. N. , Long, R. A. , Heffelfinger, J. R. , Bleich, V. C. , Krausman, P. R. , Bowyer, R. T. , Shannon, J. M. , Klaver, R. W. , Brewer, C. E. , Cox, M. , Holland, A. A. , Hubbs, A. , Lehman, C. P. , Muir, J. D. , Sterling, B. , & Monteith, K. L. (2019). Hunting and mountain sheep: Do current harvest practices affect horn growth? Evolutionary Applications, 12(9), 1823–1836. 10.1111/eva.12841 31548860 PMC6752155

[ece38692-bib-0035] Loison, A. , Festa‐Bianchet, M. , Gaillard, J. M. , Jorgenson, J. T. , & Jullien, J. M. (1999). Age‐specific survival in five populations of ungulates: evidence of senescence. Ecology, 80(8), 2539–2554. 10.1890/0012-9658(1999)080[2539:ASSIFP]2.0.CO;2

[ece38692-bib-0060] Mander, F. (2021). Facing Climate Change: The Case of the Arctic Tern (*Sterna paradisaea*). University of Washington Libraries. https://digital.lib.washington.edu:443/researchworks/handle/1773/46962

[ece38692-bib-0036] Martin, A. M. , Festa‐Bianchet, M. , Coltman, D. W. , & Pelletier, F. (2016). Demographic drivers of age‐dependent sexual selection. Journal of Evolutionary Biology, 29(7), 1437–1446. 10.1111/jeb.12883 27090379

[ece38692-bib-0059] Mehner, T. (2014). Partial diel vertical migration of sympatric vendace (Coregonus albula) and Fontane cisco (Coregonus fontanae) is driven by density dependence (1st ed., vol. 72, pp. 116–124). NRC Research Press. 10.1139/cjfas-2014-0009

[ece38692-bib-0037] Merkle, J. A. , Monteith, K. L. , Aikens, E. O. , Hayes, M. M. , Hersey, K. R. , Middleton, A. D. , Oates, B. A. , Sawyer, H. , Scurlock, B. M. , & Kauffman, M. J. (2016). Large herbivores surf waves of green‐up during spring. Proceedings of the Royal Society B‐Biological Sciences, 283(1833), 1–8. 10.1098/rspb.2016.0456 PMC4936031 27335416

[ece38692-bib-0038] Merkle, J. A. , Sawyer, H. , Monteith, K. L. , Dwinnell, S. P. H. , Fralick, G. L. , & Kauffman, M. J. (2019). Spatial memory shapes migration and its benefits: evidence from a large herbivore. Ecology Letters, 22(11), 1797–1805. 10.1111/ele.13362 31412429

[ece38692-bib-0039] Middleton, A. D. , Kauffman, M. J. , McWhirter, D. E. , Cook, J. G. , Cook, R. C. , Nelson, A. A. , Jimenez, M. D. , & Klaver, R. W. (2013). Animal migration amid shifting patterns of phenology and predation: Lessons from a Yellowstone elk herd. Ecology, 94(6), 1245–1256. 10.1890/11-2298.1 23923485

[ece38692-bib-0041] Morrissey, M. B. , Hubbs, A. , & Festa‐Bianchet, M. (2021). Horn growth appears to decline under intense trophy hunting, but biases in hunt data challenge the interpretation of the evolutionary basis of trends. Evolutionary Applications, 14(6), 1519–1527. 10.1111/eva.13207 34178101 PMC8210800

[ece38692-bib-0042] Nathan, R. , Getz, W. M. , Revilla, E. , Holyoak, M. , Kadmon, R. , Saltz, D. , & Smouse, P. E. (2008). A movement ecology paradigm for unifying organismal movement research. Proceedings of the National Academy of Sciences of the United States of America, 105(49), 19052–19059. 10.1073/pnas.0800375105 19060196 PMC2614714

[ece38692-bib-0043] Pelletier, F. (2005). Foraging time of rutting bighorn rams varies with individual behavior, not mating tactic. Behavioral Ecology, 16(1), 280–285. 10.1093/beheco/arh162

[ece38692-bib-0044] Pelletier, F. , & Festa‐Bianchet, M. (2004). Effects of body mass, age, dominance and parasite load on foraging time of bighorn rams, *Ovis canadensis* . Behavioral Ecology and Sociobiology, 56(6), 546–551. 10.1007/s00265-004-0820-7

[ece38692-bib-0045] Pelletier, F. , & Festa‐Bianchet, M. (2006). Sexual selection and social rank in bighorn rams. Animal Behavior, 71(3), 649–655. 10.1016/j.anbehav.2005.07.008

[ece38692-bib-0046] Pelletier, F. , Festa‐Bianchet, M. , Jorgenson, J. T. , Feder, C. , & Hubbs, A. (2014). Can phenotypic rescue from harvest refuges buffer wild sheep from selective hunting? Ecology and Evolution, 4(17), 3375–3382. 10.1002/ece3.1185 25535554 PMC4228612

[ece38692-bib-0047] Pelletier, F. , Hogg, J. T. , & Festa‐Bianchet, M. (2006). Male mating effort in a polygynous ungulate. Behavioral Ecology and Sociobiology, 60(5), 645–654. 10.1007/s00265-006-0208-y

[ece38692-bib-0048] Perperoglou, A. , Sauerbrei, W. , Abrahamowicz, M. , & Schmid, M. (2019). A review of spline function procedures in R. BMC Medical Research Methodology, 19(1), 19–46. 10.1186/s12874-019-0666-3 30841848 PMC6402144

[ece38692-bib-0049] Pigeon, G. , Festa‐Bianchet, M. , Coltman, D. W. , & Pelletier, F. (2016). Intense selective hunting leads to artificial evolution in horn size. Evolutionary Applications, 9(4), 521–530. 10.1111/eva.12358 27099619 PMC4831456

[ece38692-bib-0050] Poisson, Y. , Festa‐Bianchet, M. , & Pelletier, F. (2020). Testing the importance of harvest refuges for phenotypic rescue of trophy‐hunted populations. Journal of Applied Ecology, 57(3), 526–535. 10.1111/1365-2664.13562

[ece38692-bib-0051] Puckett, B. J. , & Eggleston, D. B. (2016). Metapopulation dynamics guide marine reserve design: Importance of connectivity, demographics, and stock enhancement. Ecosphere, 7(6), 1–23. 10.1002/ecs2.1322

[ece38692-bib-0057] R Core Team . (2021). R: A language and environment for statistical computing. R Foundation for Statistical Computing. https://www.R‐project.org/

[ece38692-bib-0052] Ritchot, Y. , Festa‐Bianchet, M. , Coltman, D. , & Pelletier, F. (2021). Determinants and long‐term costs of early reproduction in males of a long‐lived polygynous mammal. Ecology and Evolution, 11(11), 6829–6845. 10.1002/ece3.7530 34141259 PMC8207375

[ece38692-bib-0053] Ruckstuhl, K. E. (1998). Foraging behaviour and sexual segregation in bighorn sheep. Animal Behaviour, 56(1), 99–106. 10.1006/anbe.1998.0745 9710466

[ece38692-bib-0054] Shaw, A. K. , & Levin, S. A. (2011). To breed or not to breed: A model of partial migration. Oikos, 120(12), 1871–1879. 10.1111/j.1600-0706.2011.19443.x

[ece38692-bib-0055] Tenhumberg, B. , Tyre, A. J. , Pople, A. R. , & Possingham, H. P. (2004). Do harvest refuges buffer kangaroos against evolutionary responses to selective harvesting? Ecology, 85(7), 2003–2017. 10.1890/03-4111

[ece38692-bib-0056] Vehtari, A. , Gelman, A. , & Gabry, J. (2020). Loo: Efficient leave‐one‐out cross‐validation for fitted Bayesian models. R package version 2.4.1. https://mc‐stan.org/loo/

