# Supplementary material for: Breeding migrations by bighorn sheep males are driven by mating opportunities
Source: Ecol Evol. 2022 Mar 6;12(3):e8692. doi: 10.1002/ece3.8692 (PMC8928905; doi:10.1002/ece3.8692)
Supplement: Supplementary file 1 — Supplementary Material [file ECE3-12-e8692-s001.docx]

**Supplementary Material**

**Supplementary figures**

| a)  **** | b)  **** |
| --- | --- |

Figure S1: a) Annual number and b) age distribution of non-resident and resident bighorn males during the rut, Sheep River Provincial Park, 2000-2005, Alberta, Canada. Labels on top of bars represent proportions of non-residents by year and age. Non-resident males are not born to Sheep River females and are only present for the rut.

Figure S2: Duration of absence periods (consecutive number of days of non-observation) by individual-year for sedentary males (resident individuals present for the rut), between the 1^st^ of October and the end of the seasonal study period, Sheep River Provincial Park, 2000-2005, Alberta, Canada.

| a)   | b)   | c)   |
| --- | --- | --- |

Figure S3: Effects of a) standardised rank, b) number of competitors (number of males aged 2 years or older in the pre-rut), and c) populational sex ratio (females 2 years or older over males 2 years or older in the pre-rut) of resident bighorn males on the probability of migratory rutting tactic, Sheep River Provincial Park, 2000-2005, Alberta, Canada. Regressions lines represent estimates from the final model with other non-focal variables set to their mean value (e.g., rank at 0.49 in panels b and c). The final model was fitted with a reduced data set (142 presence/absence observations of 55 different rankable males with information on dominance). Points represent observed proportions of migrants.

**Supplementary tables**

Table S1: Annual sample size by male residency, rutting tactics and movement patterns, Sheep River Provincial Park, 2000-2005, Alberta, Canada. Resident and non-resident males are respectively those that spend most of their time in the study site and temporally immigrate for the mating season; migrant and sedentary males are residents that are respectively present and absent for the rut; itinerant and stationary males are non-reported dead sedentary resident males that respectively leave at least once (with round trips defined as absence periods greater than 6 days) and stay in the study site.

| Year | Males | Resident/Non-resident | Migrant/Sedentary | Itinerant/Stationary |
| --- | --- | --- | --- | --- |
| 2000 | 32 | 32/0 | 12/20 | 13/6 |
| 2001 | 35 | 32/3 | 15/17 | 9/7 |
| 2002 | 31 | 25/6 | 15/10 | 4/6 |
| 2003 | 26 | 20/6 | 3/17 | 14/2 |
| 2004 | 32 | 25/7 | 13/12 | 5/3 |
| 2005 | 35 | 26/9 | 18/8  - | 8/0 |

Table S2: Dates, length of study period (in days), percentage of sampling days (including days dedicated to searches) and percentage of observation days (including days dedicated to searches and with at least a census of one male group), Sheep River Provincial Park, 2000-2005, Alberta, Canada.

| Year | Study period | Length of study period | % of sampling days | % of observation days |
| --- | --- | --- | --- | --- |
| 2000 | 01/10-12/12 | 73 | 0.84 | 0.82 |
| 2001 | 01/10-13/12 | 74 | 0.84 | 0.84 |
| 2002 | 01/10-11/12 | 72 | 0.99 | 0.99 |
| 2003 | 01/10-18/12 | 79 | 0.94 | 0.91 |
| 2004 | 01/10-03/12 | 64 | 0.89 | 0.89 |
| 2005 | 01/10-08/12 | 69 | 0.93 | 0.92 |

Table S3: Candidate models fitted to explore the effects of age and demographic parameters on the probability of migratory rutting tactic, Sheep River Provincial Park, 2000-2005, Alberta, Canada. A checkmark indicates that the variable was included in the model. Symbols "+.", "+Age*" and "+Age^2^*" represent respectively that the variable is included in the model as an additive term, an interactive term with age or an interactive term with squared age. All candidate models included also sampling effort as fixed variable, and male identity and year as random variables. These candidate models were fitted separately and identically with standardised rank as individual variable instead of age and with a reduced data set (including only rankable males with information on dominance).

|  | Model variables | | | | | | | | | | |
| --- | --- | --- | --- | --- | --- | --- | --- | --- | --- | --- | --- |
|  | Individual | | Demographic | | | | | | | | |
|  | Age | Age^2^ | Number of competitors | | | Male age structure | | | Populational sex ratio | | |
| Model | +. | +. | +. | +Age*. | +Age^2^ *. | +. | +Age*. | +Age^2^ *. | +. | +Age*. | + Age^2^ *. |
| 1 |  |  |  |  | ✓ |  |  | ✓ |  |  | ✓ |
| 2 |  | ✓ |  |  | ✓ |  |  | ✓ | ✓ |  |  |
| 3 |  | ✓ | ✓ |  |  |  |  | ✓ |  |  | ✓ |
| 4 |  | ✓ |  |  | ✓ | ✓ |  |  |  |  | ✓ |
| 5 |  |  |  |  | ✓ |  |  | ✓ |  |  |  |
| 6 |  |  |  |  |  |  |  | ✓ |  |  | ✓ |
| 7 |  |  |  |  | ✓ |  |  |  |  |  | ✓ |
| 8 |  | ✓ | ✓ |  |  |  |  | ✓ | ✓ |  |  |
| 9 |  | ✓ |  |  | ✓ | ✓ |  |  | ✓ |  |  |
| 10 |  | ✓ | ✓ |  |  | ✓ |  |  |  |  | ✓ |
| 11 |  |  |  |  |  |  |  | ✓ |  |  |  |
| 12 |  |  |  |  | ✓ |  |  |  |  |  |  |
| 13 |  |  |  |  |  |  |  |  |  |  | ✓ |
| 14 |  | ✓ | ✓ |  |  | ✓ |  |  | ✓ |  |  |
| 15 |  | ✓ | ✓ |  |  | ✓ |  |  |  |  |  |
| 16 |  | ✓ |  |  |  | ✓ |  |  | ✓ |  |  |
| 17 |  | ✓ | ✓ |  |  |  |  |  | ✓ |  |  |
| 18 |  | ✓ |  |  |  | ✓ |  |  |  |  |  |
| 19 |  | ✓ | ✓ |  |  |  |  |  |  |  |  |
| 20 |  | ✓ |  |  |  |  |  |  | ✓ |  |  |
| 21 |  | ✓ |  |  |  |  |  |  |  |  |  |
| 22 |  |  |  |  |  | ✓ |  |  |  |  |  |
| 23 |  |  | ✓ |  |  |  |  |  |  |  |  |
| 24 |  |  |  |  |  |  |  |  | ✓ |  |  |
| 25 |  |  |  | ✓ |  |  | ✓ |  |  | ✓ |  |
| 26 | ✓ |  |  | ✓ |  |  | ✓ |  | ✓ |  |  |
| 27 | ✓ |  | ✓ |  |  |  | ✓ |  |  | ✓ |  |
| 28 | ✓ |  |  | ✓ |  | ✓ |  |  |  | ✓ |  |
| 29 |  |  |  | ✓ |  |  | ✓ |  |  |  |  |
| 30 |  |  |  |  |  |  | ✓ |  |  | ✓ |  |
| 31 |  |  |  | ✓ |  |  |  |  |  | ✓ |  |
| 32 | ✓ |  | ✓ |  |  |  | ✓ |  | ✓ |  |  |
| 33 | ✓ |  |  | ✓ |  | ✓ |  |  | ✓ |  |  |
| 34 | ✓ |  | ✓ |  |  | ✓ |  |  |  | ✓ |  |
| 35 |  |  |  |  |  |  | ✓ |  |  |  |  |
| 36 |  |  |  | ✓ |  |  |  |  |  |  |  |
| 37 |  |  |  |  |  |  |  |  |  | ✓ |  |
| 38 | ✓ |  | ✓ |  |  | ✓ |  |  | ✓ |  |  |
| 39 | ✓ |  | ✓ |  |  | ✓ |  |  |  |  |  |
| 40 | ✓ |  |  |  |  | ✓ |  |  | ✓ |  |  |
| 41 | ✓ |  | ✓ |  |  |  |  |  | ✓ |  |  |
| 42 | ✓ |  |  |  |  | ✓ |  |  |  |  |  |
| 43 | ✓ |  | ✓ |  |  |  |  |  |  |  |  |
| 44 | ✓ |  |  |  |  |  |  |  | ✓ |  |  |
| 45 | ✓ |  |  |  |  |  |  |  |  |  |  |
| 46 |  |  |  |  |  | ✓ |  |  |  |  |  |
| 47 |  |  | ✓ |  |  |  |  |  |  |  |  |
| 48 |  |  |  |  |  |  |  |  | ✓ |  |  |

Table S4: Model selection to explore the effects of age and demographic parameters on the probability of migratory rutting tactic, Sheep River Provincial Park, 2000-2005, Alberta, Canada. Only the 10 candidate models with the lowest LOOIC are presented. Each model was identified with the number in the first column, by which associated formula can be found in Table S3. Models were fitted with the complete data set (160 presence/absence observations of 62 different males).

| Model | LOOIC | Δ LOOIC |
| --- | --- | --- |
| 17 | 173.55 | 0.00 |
| 14 | 175.67 | 2.12 |
| 21 | 175.69 | 2.14 |
| 20 | 176.01 | 2.46 |
| 19 | 176.49 | 2.94 |
| 18 | 176.61 | 3.06 |
| 16 | 177.31 | 3.76 |
| 15 | 177.59 | 4.04 |
| 11 | 177.84 | 4.29 |
| 8 | 179.27 | 5.72 |

Table S5: Model selection to explore the effects of age, standardised rank and demographic parameters on the probability of migratory rutting tactic, Sheep River Provincial Park, 2000-2005, Alberta, Canada. Only the 10 candidate models with the lowest LOOIC are presented. Each model included either age or rank as specified in the first column and was identified with the number in the second column, by which associated formula can be found in Table S3. Models were fitted with a reduced data set (142 presence/absence observations of 55 different rankable males with information on dominance).

| Individual variable | Model | LOOIC | Δ LOOIC |
| --- | --- | --- | --- |
| Age | 21 | 151.95 | 0.00 |
| Age | 19 | 151.98 | 0.03 |
| Age | 15 | 152.03 | 0.08 |
| Age | 18 | 152.09 | 0.14 |
| Age | 17 | 152.54 | 0.59 |
| Age | 20 | 152.67 | 0.72 |
| Age | 16 | 153.02 | 1.07 |
| Age | 14 | 154.23 | 2.28 |
| Standardised rank | 17 | 155.54 | 3.59 |
| Standardised rank | 21 | 156.30 | 4.35 |

Table S6: Coefficients (on the logit scale) with corresponding 95% confidence intervals, bulk effective sample size (ESS) and tail ESS of fixed variables of the final model assessing the determinants of the probability of migratory rutting tactic and including standardised rank as individual variable, Sheep River Provincial Park, 2000-2005, Alberta, Canada. Estimates are from the final model including male identity and year as random variables. The model was fitted with a reduced data set (142 presence/absence observations of 55 different rankable males with information on dominance).

| Variable | Coefficient | 95% CI | Bulk ESS | Tail ESS |
| --- | --- | --- | --- | --- |
| Intercept | -9.40 | [-31.24; 12.04] | 14978 | 11302 |
| Standardized rank | 10.01 | [4.08; 17.44] | 15085 | 14587 |
| Standardized rank *^2^* | 4.62 | [1.60; 8.39] | 13230 | 13644 |
| Number of competitors | 0.34 | [-0.07; 0.79] | 14626 | 12150 |
| Populational sex ratio | -9.08 | [-16.68; -2.57] | 14610 | 13235 |
| Sampling effort | 0.03 | [-0.18; 0.24] | 16028 | 11634 |

Table S7: Yearly resighting rates of the 37 apparent migrant resident males by the following spring (May) or fall (October), Sheep River Provincial Park, 2000-2004, Alberta, Canada. There were no censuses in 2006.

| Year | Next spring resight rate | Next fall resight rate |
| --- | --- | --- |
| 2000 | 0.75 | 0.83 |
| 2001 | 0.33 | 0.73 |
| 2002 | 0.73 | 0.67 |
| 2003 | 1.00 | 1.00 |
| 2004 | No census | 0.92 |
